# Proteomic Response of *Deinococcus radiodurans* to Short-Term Real Microgravity during Parabolic Flight Reveals Altered Abundance of Proteins Involved in Stress Response and Cell Envelope Functions

**DOI:** 10.3390/life12010023

**Published:** 2021-12-24

**Authors:** Karlis Arturs Moors, Emanuel Ott, Wolfram Weckwerth, Tetyana Milojevic

**Affiliations:** 1 Space Biochemistry Group, Department of Biophysical Chemistry, University of Vienna, 1090 Vienna, Austria; k.moors@iem.uni-kiel.de (K.A.M.); emanuel.ott@univie.ac.at (E.O.); 2 Department of Ecogenomics and Systems Biology, University of Vienna, 1090 Vienna, Austria; wolfram.weckwerth@univie.ac.at; 3 Vienna Metabolomics Center (VIME), University of Vienna, 1090 Vienna, Austria

**Keywords:** parabolic flight, bacteria, microgravity, proteomics, outer space

## Abstract

Rapidly evolving space exploration makes understanding the short- and long- term effects of microgravity on humans, plants, and microorganisms an important task. The ubiquitous presence of the gravitational force has had an influence on the development of all living entities on Earth, and short- and long-term changes in perceived gravitational force can induce notable changes within cells. *Deinococcus radiodurans* is the Gram-positive bacterium that is best known for its extreme resistance to UV-C and gamma radiation, oxidation stress, and desiccation. Thus increased interest has been placed on this species in the context of space research. The present study aims to elucidate the short-term proteomic response of this species to real microgravity during parabolic flight. Overnight cultures of *D. radiodurans* were subjected to microgravity during a single parabola, and metabolic activity was quenched using methanol. Proteins were extracted and subsequently measured using HPLC nESI MS/MS. The results, such as the enrichment of the peptidoglycan biosynthesis pathway with differentially abundant proteins and altered S-layer protein abundance, suggested molecular rearrangements in the cell envelope of *D. radiodurans*. Altered abundance of proteins involved in energy metabolism and DNA repair could be linked with increased endogenous ROS production that contributes to the stress response. Moreover, changes in protein abundance in response to microgravity show similarities with previously reported stress responses. Thus, the present results could be used to further investigate the complex regulation of the remarkable stress management of this bacterium.

## 1. Introduction

As plans for future space exploration are becoming more ambitious, a better understanding of all factors affecting humans, plants, and microorganisms in space is necessary. Microgravity is an important variable in outer space and understanding the short- and long- term effects of microgravity on cellular processes will be important to minimize its negative effects on the physiology of any organism. To that end, outer space has become a coveted environment to investigate how microgravity affects living organisms. The International Space Station (ISS), orbiting in low Earth orbit (LEO) at an average altitude of 400 km, has been the location for multiple experiments aiming to simulate extra-terrestrial conditions, including microgravity [1,2,3,4,5]. The intensity of the gravitational pull experienced by every object on Earth is inversely proportionate to the square of the distance between the centre of the Earth and the centre of the object. On Earth, this acceleration force is approximately 9.81 m/s^2^ (= 1 *g*), whereas in LEO this force is diminished to 10^−3^ to 10^−6^ *g* [6]. To mimic these diminished forces, various ground-based approaches have also been developed that place an object in a simulated or real *free fall* state without leaving Earth’s atmosphere. Simulated microgravity is commonly achieved with three methods: 2-D clinostats, random positioning machines (RPM), and diamagnetic levitation, each attempting to negate the acceleration due to gravity on the surface of Earth [7]. Real microgravity is usually achieved using three methods: drop towers, sounding rockets, and parabolic flight [7,8,9,10,11,12].

The ubiquitous presence of the gravitational force has had an influence on the development of all living entities on Earth, and short- and long-term changes in perceived gravitational force can induce notable changes in living organisms. High importance has been attributed to investigating responses in plants and microorganisms due to their potential role as oxygen and food sources during future space missions. Plants exhibit substantial responses to changes in gravitational forces. Exposed to short-term microgravity during parabolic flight, *Arabidopsis thaliana* cells react by up-regulating Reactive Oxygen Species (ROS)-related and Ca^2+^ -related proteins [13], and similar results were also reported during simulated microgravity experiments [14]. In addition, simulated and real microgravity experiments showed that microgravity induces changes in *A. thaliana* protein synthesis (decrease), general stress response (increase), cell wall biosynthesis (increase), and carbohydrate metabolism, as well as signalling (decrease) and intra-cellular transport (increase) [15,16]. The effect of changes in gravitational acceleration has also been investigated in microorganisms. The photosynthesizing unicellular flagellate *Euglena gracilis*, subjected to microgravity during parabolic flight, responds similarly to *A. thaliana* [17]. In prokaryotes, multiple studies in real and simulated microgravity have been conducted to investigate the molecular response of various species. Studies show that despite their phylogenetic proximity, bacterial species can exhibit varying changes in virulence. *Escherichia coli* and *Salmonella typhimurium* have been shown to become more virulent in microgravity [2,18], whereas under simulated microgravity *Yersinia pestis* showed decreased secretion of T3SS effector proteins, which are necessary for peak virulence [19]. Additionally, Su et al. [20] showed that *Bacillus cereus* experience significant changes in their proteome after spaceflight. Overall, studies show that even closely related microorganisms exhibit a varied response to real and simulated microgravity, and only limited evidence exists supporting conserved molecular regulators [2,18,19,20]. 

*Deinococcus radiodurans* is the Gram-positive bacterium that is best known for its extreme resistance to UV-C and gamma radiation, oxidation stress, and desiccation [21,22,23]. These extreme survival abilities have garnered interest in this species in the context of space research. Recently, *D. radiodurans* survived interplanetary transfer during three years of exposure to outer space [24]. Our subsequent multi-omics study on *D. radiodurans* exposed outside the ISS in the framework of the Tanpopo orbital mission provided evidence for a multifaceted stress response to 1 year of space exposure [25]. We have also conducted simulated microgravity experiments on *D. radiodurans* using 2-D clinostats to investigate this species’ specific response to long-term changes in gravitational forces [26]. Under simulated microgravity conditions, this bacterium upregulates proteins involved in DNA stress response, carbohydrate degradation, and general gene regulation (DR_1174, DR_2299, DR_0750, DR_2419). *D. radiodurans* also exhibits decreased abundance of ribosomal proteins in response to microgravity, as observed in other bacterial species [27]. The study by Ott et al. [26] delivered a good overview of the long-term changes occurring in *D. radiodurans* due to simulated microgravity, however, it is unclear how the sheer stress and fluid convection associated with this method contributed to these changes and what the short-term effects of microgravity on this bacterium are.

In the present study, parabolic flight was used to subject microbiological samples to real microgravity. *D. radiodurans* cells were subjected to microgravity and increased gravity during parabolic flight, and their proteome was subsequently analysed with high-performance liquid chromatography/nano-electrospray ionization in tandem with mass spectrometry (HPLC/nESI-MS/MS). Overall, the aim of this study was to elucidate the changes to the *D. radiodurans* proteome that occur after exposure to short-term real microgravity and increased gravity aboard parabolic flight and to compare this response to the response previously reported in the literature. This study can contribute to understanding how cells react to reduced gravity immediately after exposure without other, more influential environmental factors present in low Earth orbit.

## 2. Materials and Methods

### 2.1. Bacterial Growth

Liquid cultures of *D. radiodurans* R1 were grown under conditions previously described in Ott et al. [26]. Briefly, *D. radiodurans* was cultivated for 15 h in TGB medium (1%(w/v) tryptone, 0.6%(w/v) beef extract, 0.2%(w/v) glucose) at 30 °C in an incubator with an agitation speed of 150 rpm until it reached the mid-exponential phase. On the day of the parabolic flight, overnight cultures were transferred into 12 syringes (2 mL of culture per syringe) belonging to the top part of the custom injection device (Figure S1). Falcon tubes, where cultures were to be injected, were filled with 10 mL of 100% methanol (MeOH). 

### 2.2. Parabolic Flight

The parabolic flight was performed from Stockerau, Austria (https://www.blufly.at/parabelflüge-1/) on 14 September 2019. Ground temperature was 28 °C and ground pressure was 1015 hPa. Parabolas were flown between 2000 ft and 4500 ft (609 m and 1371 m) above sea level, with a temperature of 19 °C estimated for 4500 ft based on temperature lapse rate [28]. The custom injection device (Figure S1) filled with the cultures of *D. radiodurans* was placed on board an MDM-1 Fox glider. After 10 s of 0 gravity (0 *g*), bacterial cultures were injected into the Falcon tubes and quenched with methanol followed by storage on dry ice (Figure S1). In parallel, ground control cultures (GC) were quenched in methanol with the same custom injection device (Figure S1) prior to the flights and kept at static 1 g control on the ground in dry ice. Flight control (FC) cultures were quenched with methanol with the custom injection device during parabolic flight before entering the zero-gravity stage, attaining on average 6.6 *g* (Figure S2). Four biological replicates of the cultures of *D. radiodurans* for each of the conditions were used.

### 2.3. Protein Extraction, Purification, and Digestion

Protein extraction, purification, and digestion was performed as described in Ott et al. [29]. Briefly, after the parabolic flight, each culture replicate was stored in 12 mL methanol/water. The samples were put in ultracentrifuge tubes and centrifuged at 50,000× *g* speed (4 °C for 15 min). The supernatant was discarded, 1 mL of ice-cold methanol was added, and the suspension was transferred to fresh Eppendorf tubes. Another centrifugation step (21,000× *g*/4 °C/15 min) was applied, and the pellet was air-dried. TRIzol was added to the pellet and the pellet was further homogenized in a bead beater (30 s, 6.5 m/s). After extraction, protein pellets were centrifuged (21,000× *g*, 15 min, 4 °C) and washed twice with 1.8 mL ice-cold methanol and once with 1.8 mL ice-cold acetone. At each wash, the pellets were ultrasonicated for 5 min and centrifuged (21,000× *g*, 15 min, 4 °C), discarding the supernatant. After the acetone wash, the pellets were air-dried. A total of 60 μg of protein from each sample was used for digestion. For the reduction step, samples were adjusted to 5 mM dithiothreitol (DTT) and incubated for 45 min at 37 °C at 700 rpm. Samples were then alkylated by adjusting the iodoacetamide (IAA) concentration to 10 mM then incubated for 60 min in darkness at RT at 700 rpm. Alkylation was stopped by adjusting the DTT concentration to 10 mM DTT. Samples were incubated for 15 min at RT. Three microliters of trypsin beads (Promega) were added to digest proteins, and samples were incubated at 37 °C at 10 rpm for 16 h. Digestion was halted by placing samples on ice, followed by desalting and a protein quantification procedure as described in Ott et al. [29].

### 2.4. HPLC nESI MS/MS

Shotgun proteomics measurements were performed as described previously by Ott et al. [29]. Briefly, 5  µL of each sample were injected into an Orbitrap Elite (Thermo Fisher Scientific, Bremen, Germany) with the following measurement settings: full scan range 350–1800 m/z resolution 120,000, max. 10 MS2 scans (activation type CID), repeat count 1, repeat duration 30 s, exclusion list size 500, exclusion duration 30 s, charge state screening enabled with rejection of unassigned and +1 charge states, minimum signal threshold 500 [30]. Raw output analysis was performed with Maxquant [31] with the following settings: 20 ppm first search peptide tolerance, 4.5 ppm main search peptide tolerance, maximum of 2 missed cleavages, maximum number of 5 modifications per peptide (variable: oxidation (M) and acetylation of protein N-term, fixed: carbamidomethylation (C)), label free quantification of samples. Protein identification was performed using the Uniprot database (release-2019_10). Minimum peptide length for identification was set to seven amino acids and one unique peptide was required for protein identification (FDR 1% based on target decoy database). 

### 2.5. Quality Control

All proteomic data processing was done in RStudio, R version 4.0.4 [32]. Raw label-free quantification (LFQ) data containing 1925 proteins was quality controlled using PCA. Sample GC_4 was removed from the analysis due to its large distance from the other GC samples. Removal of this sample decreased within-group variance and increased between-group variance in PCA. Proteins with more than one missing value in any treatment (prior to sample removal) were excluded from the analysis to improve the fidelity of imputation. 

### 2.6. Imputation and Statistical Analysis

Analysis was performed using the DEP R package, version 1.10.0 [33]. This pipeline uses variance stabilizing normalization (vsn) on the proteomics data, which has been shown to be appropriate and to perform well on label-free quantification proteomics data [34]. After normalization, the built-in DEP functionality was used for quantile regression-based left-censored function (QRILC) imputation of the missing data, as suggested by the authors. For statistical analysis, DEP uses the comprehensive and commonly used *limma* R package [35]. *Limma* is an R/Bioconductor package that provides a pipeline for analyzing gene expression experiment data using linear models. Despite being initially developed for RNA microarray data analysis, studies have shown that *limma* can also be successfully used on proteomics data [36,37]. A standalone *p*-value adjustment was performed using the *q*-value R package. The often-used *p*-value correction method Benjamini–Hochberg (also known as FDR correction) assumes that all *p*-values are evenly distributed and thus come from a null distribution. However, the *p*-value distribution likely contains a mixture of *p*-values from the alternative and null distributions. The *q*-value package estimates the true proportion of null *p*-values, then obtains the *q*-value based on this new information.

### 2.7. Functional Annotation and Gene Ontology Enrichment Analysis

Automatic proteins functional annotation was performed using the DAVID functional annotation tool [38,39]. Proteins with no positive DAVID hits (which occurred due to outdated database version or general lack of information) were manually annotated using the respective UniProt Gene Ontology annotation or information from the relevant literature [40]. Gene ontology (GO), biological process, and molecular function enrichment analyses (Fisher’s exact test and FDR correction; alpha = 0.05) were performed on the differentially abundant proteins with the online GO enrichment tool [41], as it contains the most up-to-date GO annotation. Proteins with increased and decreased abundance were considered separately. 

### 2.8. KEGG Pathways

KEGG (Kyoto Encyclopaedia of Genes and Genomes) Pathway enrichment [42] was obtained using the STRING DB analysis tools [43]. Each grouping/cluster was input into the online protein–protein interaction database, and pathways significantly enriched (<0.05 FDR) with the present proteins were considered for further investigation. Proteins with increased and decreased abundance were considered separately when using this tool. Pathways were visualized in Cytoscape [44], and figure illustrations were created with biorender.com (accessed on 19 December 2021).

## 3. Results

### 3.1. Filtering, Quality Control, and Imputation

**Figure 1:**
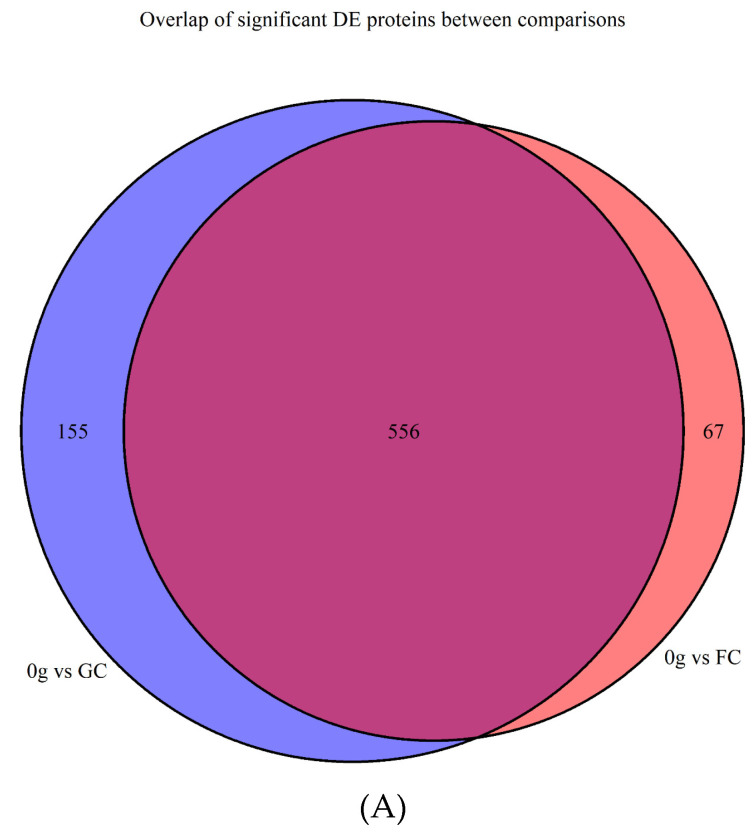

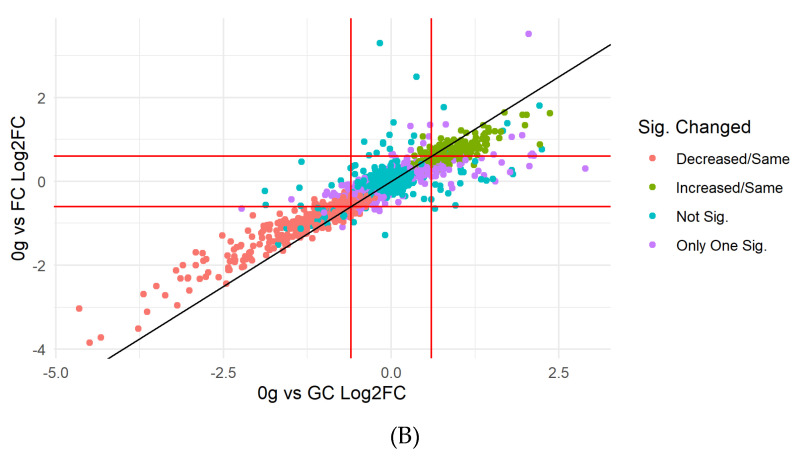
Comparison of the ‘’0 *g* vs. GC’’ and ‘’0 *g* vs. FC’’ contrasts of *D. radiodurans* proteins based on proteomics data obtained during parabolic flight. (**A**) Venn diagram of significantly changed proteins in the two contrasts. (**B**) Scatter plot of ‘’0 *g* vs. FC’’ vs. ‘’0 *g* vs. GC’’ Log2 fold changes of all 1200 proteins. Decreased/Same—protein was decreased in abundance in 0 *g* in both contrasts; Not Sig.—protein showed no significant changes in both contrasts; Only One Sig.— protein was significant in one of the two contrasts; Increased/Same—protein was increased in abundance in 0 *g* in both contrasts.

**Figure 2:**
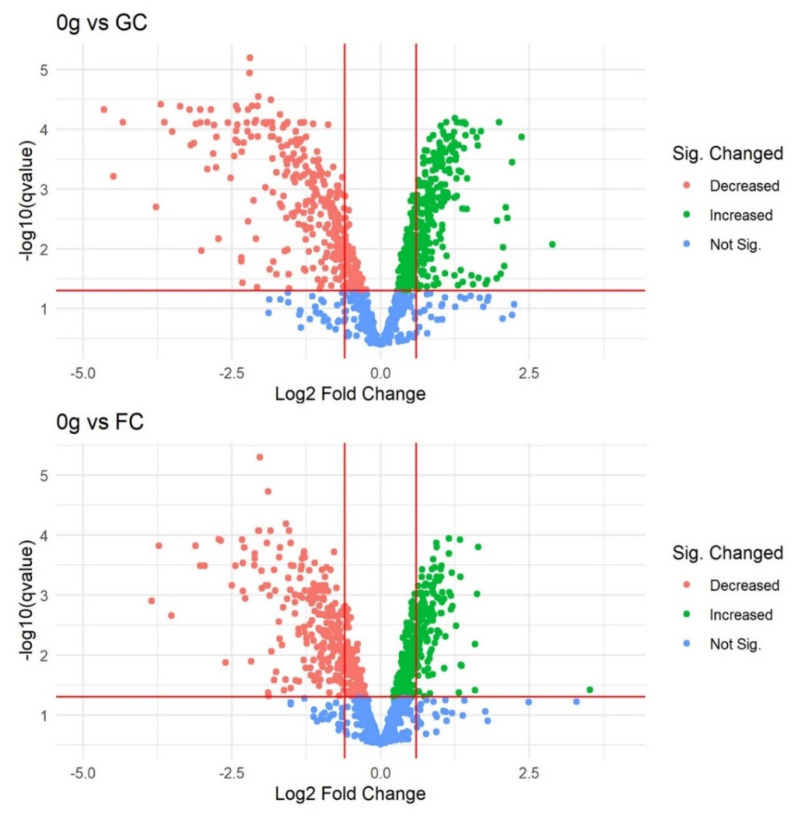
Volcano plot of *D. radiodurans* proteins in the ‘0 *g* vs. FC’ contrast based on proteomics data obtained during parabolic flight. Horizontal red line indicates significance threshold (*q*-value < 0.05); Vertical lines at Log2FC = (−0.6) and Log2FC = (0.6) represent a Fold Change of approximately −1.5 and 1.5, respectively.

A total of 1925 proteins were identified in at least one replicate, which represents 61.7% of the *D. radiodurans* genome. Three treatment groups of four replicates were present. These treatment groups represented the gravity state in which the cells were placed: 0 *g* (microgravity), flight control (FC; increased gravity), and ground control (GC; control). Data was filtered to a maximum of 1 missing value in the treatment group (at least 3/4 numerical values in each 0 *g*, FC, and GC), which decreased the number of proteins for analysis from 1925 to 1200. PCA plots, created with the DEP PCA tool, showed that sample GC_4 was an important driver for PC2, which represented the within-group separation and decreased between-group separation in PC1 (Figure S3). Based on these QC results, sample GC_4 was removed from further analysis. QC plots obtained with the built-in DEP tools showed no additional issues. After removal, imputation of remaining missing values was performed with QRILC with 10.4% (125/1200) of values being imputed. PCA of the 1200 proteins showed clear separation between the microgravity and other groups in PC1, explaining 46.9% of variation (Figure S3). Statistical analysis with the DEP pipeline showed 711 (*q*-value < 0.05) differentially abundant proteins between cells subjected to microgravity (0 *g*) and ground control (GC). A total of 357 proteins were more abundant and 354 were less abundant when subjected to microgravity during parabolic flight. Between the FC and GC samples, no significant differences were obtained. The comparison between 0 *g* and FC shows 623 differentially abundant proteins, with most proteins being differentially abundant as in the 0 *g*/GC comparison. Only 67 proteins were unique (not found in the 0 *g* vs. GC contrast) and are shown in Table 1. Figure 1 shows that there is large overlap in significant changes between the two contrasts, and that proteins with significant decreases in abundance also have on average larger Log2 FC values. Volcano plots in Figure 2, representing the two contrasts, show the differential abundances of the two contrasts graphically. All Log2 expression values, Log2 fold changes between treatments, and statistical analysis results can be found in Table S1. 

#### Differentially Abundant Protein Grouping and GO Enrichment

**Figure 3:**
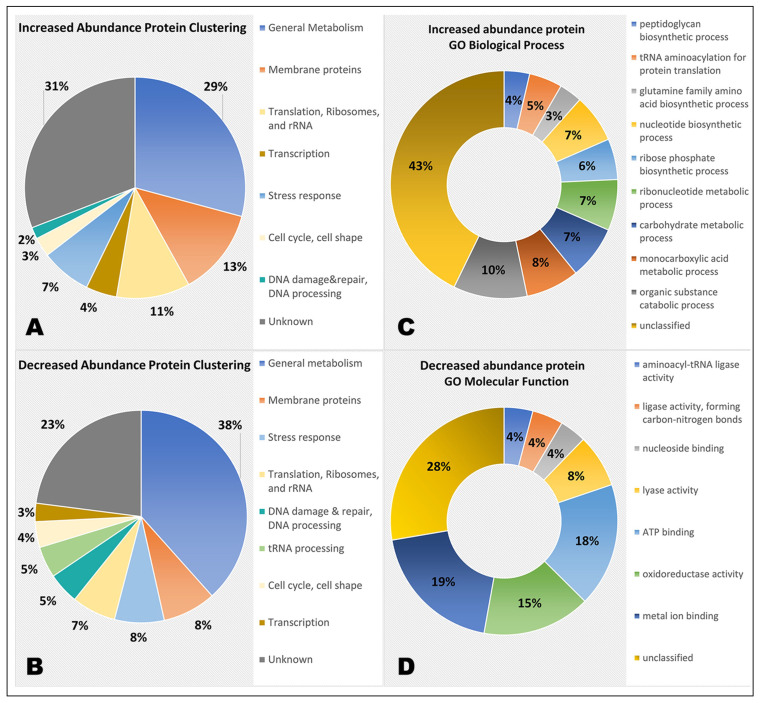
Groups/clusters of differentially abundant proteins of *D. radiodurans* based on proteomics data obtained during parabolic flight. (**A**) Groups/clusters of proteins with significantly increased abundance in the ‘0 *g* vs. GC’ contrast. (**B**) Groups/clusters of proteins with significantly decreased abundance in the ‘0 *g* vs. GC’ contrast. (**C**) GO biological process (BP) enrichment of proteins with increased abundance (0 *g* vs. GC). (**D**) GO molecular function enrichment of proteins with increased abundance (0 *g* vs. GC). For GO enrichment, the most specific enriched sub-class for each category is shown.

Differentially abundant proteins (0 *g*/GC) were grouped based on their GO biological process and molecular function tags obtained automatically with DAVID and manual assignment from UniProt. Proteins with increased and decreased abundance were grouped separately. In the increased abundance grouping, 275 proteins were placed in 8 groups while 82 proteins could not be clustered due to lack of functional information. A total of 244 proteins with significantly decreased abundance were clustered in seven groups; 109 proteins could not be grouped due to lack of information. The groups/clusters and the number of proteins in each group are shown in Figure 3, and all groups/clusters, as well as Log2 fold changes of proteins with significantly decreased abundance, are shown in Table S2. Gene ontology enrichment analysis of biological processes (BP) and molecular function (MF) were performed with the online gene ontology enrichment tool. Enriched ontology terms for proteins with increased abundance in the 0 *g* vs. GC contrast are shown in Figure 3. BP terms show enrichment in carbohydrate, ribonucleotide, and monocarboxylic acid metabolism, as well as amino acid, nucleotide, and ribose phosphate biosynthesis. Additionally, tRNA aminoacylation and peptidoglycan biosynthesis show significant enrichment. MF terms show enrichment in lyase, ligase, aminoacyl-tRNA ligase, and oxidoreductase activity, as well as metal ion, ATP, and nucleoside binding. For proteins with decreased abundance in this contrast, ‘translation’ (BP) and ‘structural constituent of ribosome’ (MF) and ‘rRNA binding’ (MF) were the only enriched terms. Overall, the enriched terms reflect the manual grouping of the proteins well.

### 3.2. General Metabolism

General metabolism was the largest group in proteins with both increased and decreased abundance. For proteins with increased abundance, general metabolism contained 137 proteins involved in various biosynthetic, as well as metabolic and catabolic, processes, such as arginine biosynthesis (KEGG dra00220); purine metabolism (KEGG dra00230); valine, leucine, and isoleucine biosynthesis (KEGG dra00290) and degradation (KEGG dra00280); and starch and sucrose metabolism (KEGG dra00500). Apart from this, proteins with increased abundance are involved in amino acid biosynthesis and iron–sulfur cluster assembly. For decreased abundance proteins, many pathways with a broad function are found, such as purine metabolism (dra00230) and carbon metabolism (dra01200). Additionally, some pathways overlap with the general metabolism pathways enriched by proteins with increased abundance, such as fatty acid biosynthesis (dra00061) and fatty acid metabolism (dra01212). All significant pathways and differentially abundant proteins belonging to them can be found in Table S3.

**Figure 4:**
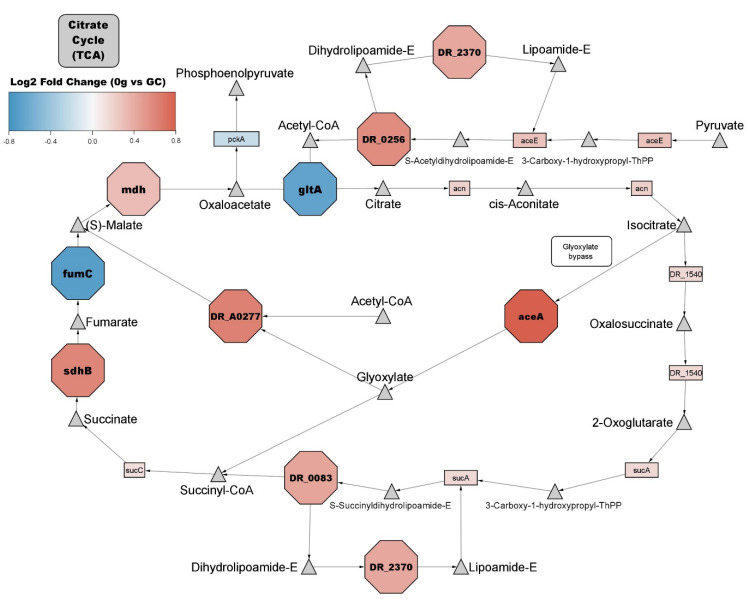
*D. radiodurans* TCA pathway. Triangles represent metabolites; rectangles represent non-significant proteins; octagons represent significantly changed proteins. Red = increased abundance proteins, blue = decreased abundance proteins of *D. radiodurans* exposed to microgravity during the parabolic flight.

Further investigation within the general metabolism group showed proteins belonging to numerous KEGG pathways involved in energy metabolism. The citric acid cycle pathway (KEGG dra00020) contains multiple proteins with significantly increased abundance (Figure 4). Most other proteinaceous members of the pathway appear to be more abundant but do not meet the significance threshold. In the pentose phosphate pathway (KEGG dra00030), five proteins show significantly increased abundance (Table 2).

### 3.3. Membrane Proteins

**Figure 5:**
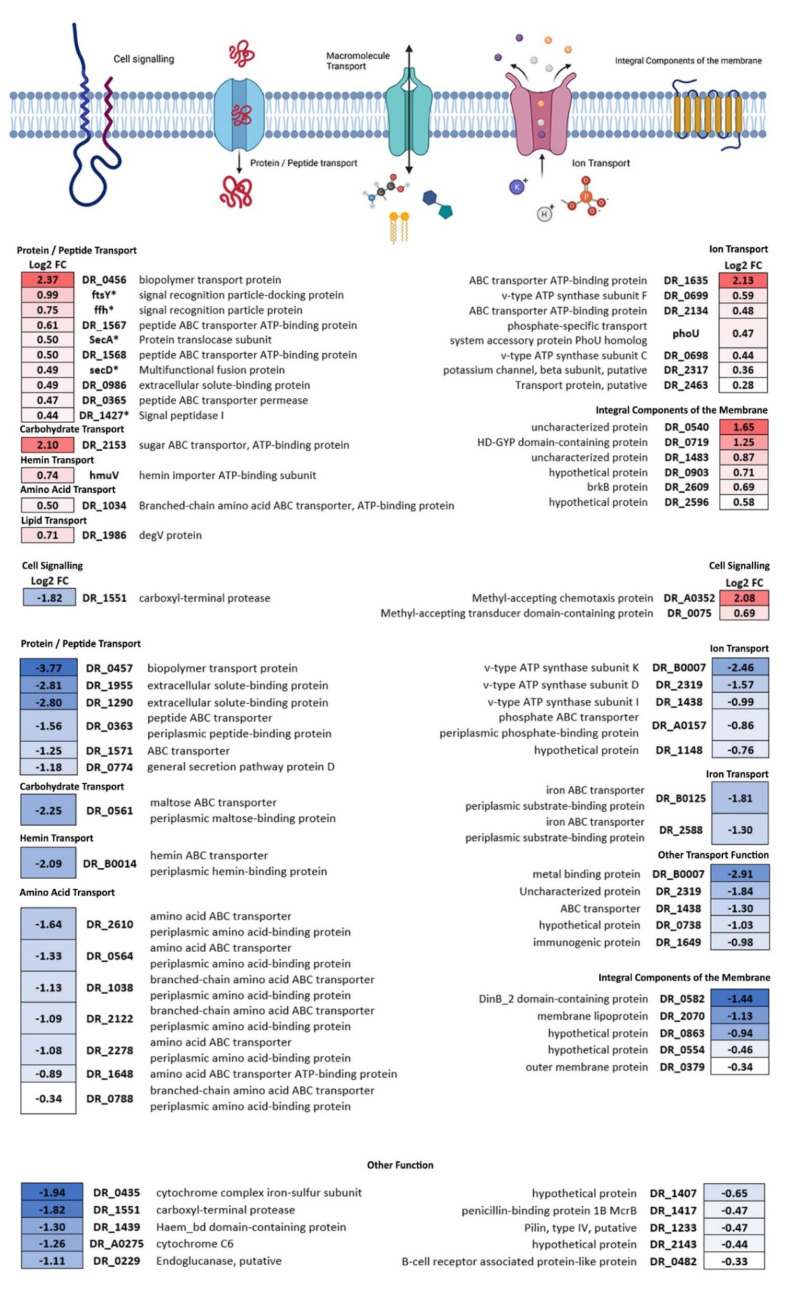
Proteins with significantly increased and decreased abundance in the membrane proteins cluster. Members of the central Sec pathway are marked with an asterisk (*). Numbers represent the Log2 fold change.

The second largest group of both proteins with increased and decreased abundance are membrane proteins. As shown in Figure 5, transport proteins represent the largest part of the increased abundance proteins in this cluster (21 out of 30 proteins), and their functions involve protein/peptide (10 proteins), ion (7 proteins), amino acid (1 protein), carbohydrate (1 protein), lipid (1 protein), and hemin transport (1 protein). Additionally, two significant proteins show involvement in cell signalling, whereas the remaining six proteins in this group are associated with the integrity of the membrane. Proteins with significantly increased abundance are enriched in three KEGG pathways: protein export (5/15 proteins, dra03060; represented with * in Figure 5), bacterial secretion system (4/11 proteins, dra03070, showing overlap with dra03060), and quorum sensing (9/58 proteins, dra02024). A total of 43 decreased abundance proteins are represented in the membrane proteins group. Transport proteins also constitute the largest proportion (28 out of 43 proteins), showing a decrease in protein/peptide (6 proteins), carbohydrate (1 protein), hemin (1 protein), ion (5 proteins), and iron (2 proteins) transporters. Notably, seven amino acid transporters show a significant decrease in abundance compared to GC, whereas only one amino acid transporter is significantly increased in abundance. The exact function of the five transport proteins (Figure 5, “Other Transport function”) is unknown. Moreover, five membrane integrity proteins were decreased in abundance in this group, whereas the exact function of 10 proteins in this group (Figure 5, “Other function”) could not be specified. Proteins with significantly decreased abundance are enriched in three KEGG pathways: ABC transporters (10/79 proteins, dra02010), oxidative phosphorylation (3/33 proteins, dra00190), and quorum sensing (5/58 proteins, dra02024). It has to be noted that the quorum sensing pathway does not correspond uniquely to *D. radiodurans* functions but is present in multiple species and *D. radiodurans* has proteins for some of the depicted reactions [45]. 

### 3.4. Translation, Ribosomes, and rRNA

**Figure 6:**
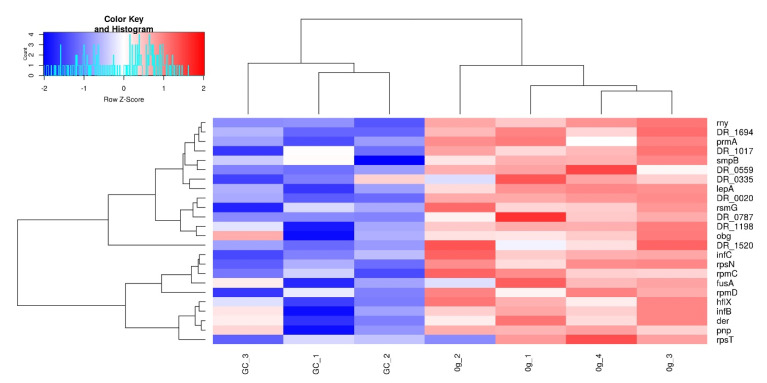
Heatmap of the *D. radiodurans* proteins with significantly increased protein abundance in the *translation, ribosomes, and rRNA* cluster. Rows are z-score normalized. Clustering based on complete linkage and Euclidean distance.

The translation, ribosomes, and rRNA cluster is the third largest in both increased abundance and decreased abundance proteins. This cluster contains proteins involved in translation, such as ribosomal proteins and other components of the translational process (Figure 6). The protein with the most significantly increased abundance is DR_1145 (GTP-binding protein LepA), showing an increase of more than threefold compared to GC. Three more proteins are increased in abundance by more than twofold, DR_0335 (ATP-dependent RNA helicase, putative), DR_0020 (ribonuclease II family protein), and DR_2109 (ribosomal protein S14). The protein with the most decreased abundance is DR_0901 (ribosome-binding factor A), showing a decrease of more than 22.5-fold compared to GC. The decreased abundance proteins have overall much higher fold-changes than the increased abundance proteins, with 29/38 proteins showing a decrease of more than twofold. Moreover, the increased abundance proteins are more heterogenous in their apparent function, whereas most of the decreased abundance proteins in this cluster are ribosomal proteins and are part of the ribosome pathway (30/53 proteins, dra03010).

### 3.5. Cell Cycle and Cell Shape

**Figure 7:**
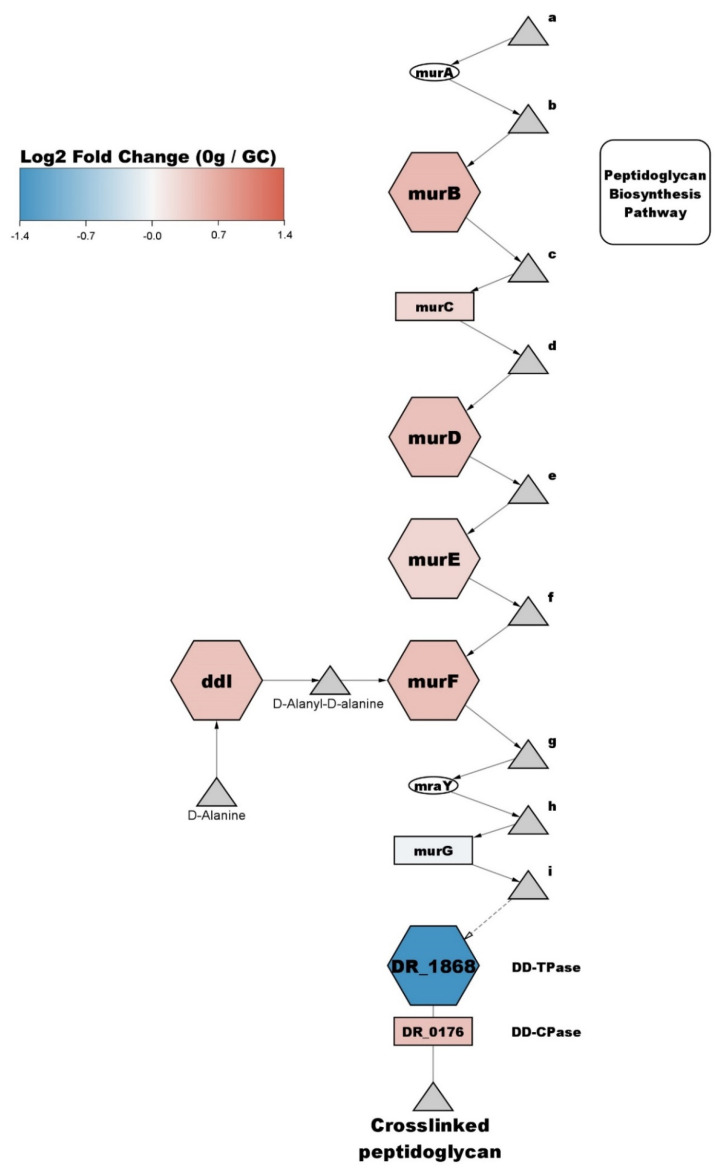
Peptidoglycan pathway of *D. radiodurans* exposed to microgravity during the parabolic flight is enriched with increased abundance proteins from the Cell cycle and cell shape cluster. Triangles represent metabolites; rectangles represent non-significant proteins; octagons represent significantly changed proteins; ovals represent unmeasured or filtered proteins. a = UDP-N-acetyl-alpha-D-glucosamine, b = UDP-N-acetyl-3-(1-carboxyvinyl)-D-glucosamine, c = UDP-N-acetylmuramate, d = UDP-N-acetylmuramoyl-L-alanine, e = UDP-N-acetylmuramoyl-L-alanyl-D-glutamate, f = UDP-N-acetylmuramoyl-L-alanyl-gamma-D-glutamyl-meso-2,6-diaminopimelate, g = UDP-N-acetylmuramoyl-L-alanyl-D-glutamyl-6-carboxy-L-lysyl-D-alanyl-D-alanine, h = undecaprenyl-diphospho-N-acetylmuramoyl-L-alanyl-D-glutamyl-meso-2,6-diaminopimeloyl-D-alanyl-D-alanine, i = undecaprenyl-diphospho-N-acetylmuramoyl-(N-acetylglucosamine)-L-alanyl-D-glutamyl-meso-2,6-diaminopimeloyl-D-alanyl-D-alanine.

Out of 14 proteins with significantly increased abundance in the cell cycle and cell shape cluster, three are increased in abundance by more than twofold, DR_1488 (membrane-bound protein LytR), DR_0770 (fimbrial assembly protein PilM), and DR_1397 (hypothetical protein). The protein with the most decreased abundance in this cluster is DR_2394 (N-acetylmuramoyl-L-alanine amidase), which shows a decrease of 12.5-fold. Protein DR_0938 (hypothetical protein) is also strongly decreased in abundance, decreasing five-fold compared to GC. Three other proteins show a decrease of more than twofold, DR_1062 (FemA-like protein), DR_1868 (penicillin-binding protein 2), and DR_0853 (gliding motility protein). Additionally, 5/14 increased abundance (ddI, murB, murD, murE, DR_0768) and 1/9 (DR_1868) decreased abundance proteins are a part of the Peptidoglycan biosynthesis pathway (KEGG dra00550), seen in Figure 7. Proteins from this cluster are present in other cell-wall related pathways, such as vancomycin resistance (KEGG dra01502) and cationic antimicrobial peptide (CAMP) resistance (KEGG dra01503).

### 3.6. tRNA-related Proteins

This cluster is only present in proteins with increased abundance and contains 17 proteins. Of these, 15 proteins are a part of the tRNA-aminoacylation pathway (KEGG dra00970). The other two proteins, DR_1150 ((dimethylallyl)adenosine tRNA methylthiotransferase) and queA (S-adenosylmethionine:tRNA ribosyltransferase-isomerase), are also the two most highly abundant proteins in this cluster, with abundance 3.2- and 2.3-fold higher than in GC, respectively. 

### 3.7. DNA Damage and Repair, DNA Processing

A total of 17 increased abundance and six decreased abundance proteins were placed in this cluster (Table 2). Increased abundance proteins are significantly enriched in multiple KEGG pathways linked with DNA repair: nucleotide excision repair (4/8 proteins, dra03420), homologous recombination (4/17 proteins, dra03440), and base excision repair (2/14 proteins, dra03410). Proteins with significantly decreased abundance were not enriched in any KEGG pathways, but one protein, DR_B0067 (extracellular nuclease), showed a 4.58-fold decrease compared to GC.

### 3.8. Transcription

Proteins involved in regulation of DNA transcription are represented in both increased abundance (10 proteins) and decreased abundance groups (16 proteins) (Table 2). The largest increase in abundance in this cluster was shown by hypothetical protein DR_1872, with nearly a four-fold increase compared to GC. Three other proteins, rho (transcription termination factor Rho), DR_0911 (DNA-directed RNA polymerase subunit beta), and DR_0200 (Lpr/AsnC family transcriptional regulator), were increased around twofold compared to GC. Increased abundance proteins from this cluster were enriched in four pathways: RNA polymerase (3/4 proteins, dra03020), pyrimidine metabolism (4/39 proteins, dra00240), purine metabolism (3/69 proteins, dra00230), and metabolic pathways (5/39 proteins, dra01100). Conversely, protein DR_0907 (CSD family cold shock protein) showed the strongest decrease compared to GC, decreasing in abundance approximately 25-fold. DR_A0065 (DNA-binding protein HB) and DR_2415 (DNA-binding response regulator) decreased in abundance by nine-and eight-fold, respectively, and four other proteins decreased in abundance by twofold or more. Decreased abundance proteins were not present in any enriched KEGG pathways. 

### 3.9. Stress Response

The stress response group includes proteins that have previously been linked to significant changes under stress conditions, thus annotated as such through GO terms or UniProt descriptions (e.g., “response to osmotic stress”, “response to heat”, “response to antibiotic”, “response to oxidative stress”, “oxidoreductase activity” (linked to oxidative stress)). This cluster was further grouped according to the type of stress response [46]: general stress response, oxidative stress response, and osmotic stress response. General stress response works to protect the cell and restore damage to intracellular structures, such as proteins and the cell envelope. In this group, numerous proteases are differentially abundant, including well-known stress response proteins ClpB, GrpE, and Lon protease (Table 2). The oxidative stress response aims to reduce ROS-induced damage through ROS scavenging and maintaining redox homeostasis within the cell. Thus, this group contains multiple proteins with oxidoreductase activity and is involved in ROS-neutralization (Table 2). The osmotic stress response regulates the osmotic environment within the cell and results showed two proteins with significantly altered abundance—the increased abundance DR_1829 (HAMP domain-containing protein), and the decreased abundance DR_1538 (osmotically inducible protein C) (Table 2).

## 4. Discussion

The aim of the present study was to elucidate the molecular events in *D. radiodurans* that occur in response to short-term real microgravity and increased gravity that are achieved using parabolic flight. Results showed that, compared to ground controls, microgravity had a pronounced effect on protein abundance. Increased gravity did not elicit a response that significantly induced changes in the proteome of *D. radiodurans*. The results of these analyses are discussed below, followed by suggestions for future research and a conclusion.

### 4.1. Changes in General Metabolism Reflect Previously Reported Stress Response

The largest proportion of the proteins of *D. radiodurans* with significantly changed abundance were clustered in the *general metabolism* group, which incorporates proteins that have functions in the metabolism and catabolism of amino acids, lipids, carbohydrates, proteins, and other metabolites as well as energy (ATP) production. Multiple KEGG pathways were significantly enriched with proteins from the *general metabolism* cluster. Some broad pathways, such as “carbon metabolism (dra01200)”, “biosynthesis of antibiotics (dra01130)”, and “biosynthesis of secondary metabolites (dra01110)” were significantly enriched with proteins with significantly increased abundance. Several more specific pathways were also significantly enriched and could offer an insight into the specific processes involved in the immediate response to microgravity. Many of the pathways enriched with proteins with significantly increased abundance are involved in energy metabolism, such as “citrate cycle” (TCA cycle) (dra00020) and “pentose phosphate pathway” (dra00030). These results align very well with previously reported *D. radiodurans* responses to oxidative stress, as summarized in the comprehensive review by [22]. It has been shown that *D. radiodurans* utilizes proteolysis as the main energy source to obtain amino acids that are the preferred carbon source in these bacteria [47]. The present results also show many increased abundance proteins in the *general metabolism* cluster to be involved in amino acid biosynthesis and metabolism pathways. Previous studies have suggested that a microgravity environment lacks the same physical forces as 1g gravity, which reduces metabolite exchange with the cell, limiting the exchange to diffusive processes [48,49]. This state has been shown to result in the overexpression of genes related to starvation and energy demand [50], and the present results could reflect an increased energy requirement due to changes in gravity-controlled physical forces. In addition, stress induced by ionizing radiation has been shown to induce proteolytic activity inside these bacteria and is thought to be a way to decrease the biosynthetic (thus energy) requirements during post-irradiation recovery [51,52]. It is also believed that *D. radiodurans* imports degraded proteins from the extracellular milieu to aid in recovery, which could be mediated by secreted subtilisin-like serine proteases and increased protein/peptide/amino acid transport across the membrane [51,52]. In the present research, various protein-folding proteins as well as membrane proteins involved in protein/peptide import and other macromolecule import are increased in abundance (Figure 5 and Table 2). Among those are chaperone ClpB, GrpE, and Lon proteases. Clp family proteases and chaperones are important components of the general stress response in bacteria, preventing protein misfolding and aggregation, and degrading misfolded proteins [46,53]. Lon proteases have been linked with the general stress response in *D. radiodurans*, increasing in abundance in severe stress conditions [30]. GrpE is known as a regulator of protein-folding machinery in *D. radiodurans* [22] and likely aids in the stress response induced by microgravity. Additionally, protein DR_0456, which bears similarity to the *E. coli* exbB/tolQ family involved in biopolymer import and membrane stability [54], is strongly induced in microgravity. Overall, these results suggest that microgravity could be causing *D. radiodurans* to accumulate differentially abundant proteins involved in amino acid biosynthesis, as well as protein processing, transport, and general stress response.

Alongside proteolysis, glucose metabolism is also important for recovery from stress conditions as it provides metabolites that are used for DNA repair and ROS scavenging [55]. The pentose phosphate pathway (PPP) shows multiple proteins with significantly increased abundance, including G6PDH (gene name *zwf*), which is an important protein in this pathway (1.74-fold increase in microgravity). This protein has high constitutive expression, being expressed four times higher in *D. radiodurans* than *E. coli* under normal conditions [55], and G6PDH mutants show decreased tolerance to stressors like hydrogen peroxide and UV light [56]. These results might indicate that the PPP and G6PDH plays an important role in the response to changes in gravitational forces. Additionally, *D. radiodurans* have been shown to modulate the activity within the TCA cycle to prioritize the glyoxylate bypass when grown in a defined minimal medium or in response to radiation [51,52]. The glyoxylate bypass (A.K.A. glyoxylate shunt) is known to be an important modulator for the use of acetate and fatty acids in gluconeogenesis [57]. Moreover, research has indicated that the glyoxylate bypass plays a role in increased virulence and resistance to oxidative stress in fungi and bacteria [58,59]. It has been shown that an important source of ROS comes from the self-oxidation of respiratory chain enzymes where electrons are transferred from NADH and FADH_2_ to oxygen [60]. The glyoxylate bypass, seen in Figure 4, converts isocitrate directly into glyoxylate and succinate, deprioritising two NADH producing reactions, thus potentially decreasing the amount of ROS produced [51,52]. In the present results, members of the glyoxylate bypass show significant increases in abundance, whereas the TCA cycle reactions that are deprioritized show no significant change. Thus, these results might indicate that elevated pull of the glyoxylate bypass’ enzymes could potentially help us cope with ROS during the long-term effects of exposure to microgravity.

### 4.2. Cell Envelope Processes Are Affected by Microgravity

*D. radiodurans*, although staining Gram-positive [61], has a cell envelope structure similar to that of Gram-negative bacteria: inner and outer membranes containing a layer of peptidoglycan and the periplasmic space between the two [62,63]. The outer membrane is a mixture of lipids followed by the hexagonally packed surface layer composed of HPI (hexagonally packed intermediate-layer surface protein). This outer-most mixture of HPI, SlpA and other components is often referred to as the S-layer, and such a layer is also present in other bacterial and archaeal species [64]. Previous research has shown that SlpA is of paramount importance in cell envelope integrity, and SlpA mutants showed decreased resistance to solvents and sheer stress, whereas HPI mutations had little to no impact on this aspect [65]. This importance of SlpA is interpreted by Misra et al. [66], who, based on their results, proposed that SlpA attaches to HPI on one end and to the peptidoglycan layer on the other, providing a structural role and actually making up most of the periplasmic space. A recent study by Farci et al. [67] showed that the actual composition of the *D. radiodurans* S-layer might be more complicated than initially thought, consisting not only of SlpA but a range of other proteins. They also provided evidence that SlpA might be a part of a larger protein complex called S-layer deinoxanthin-binding complex (SDBC) based on observations of SlpA binding carotenoid deinoxanthin, a strong protective antioxidant [68]. Upon analysis of the SDBC, it was shown that it consists of multiple proteins, namely, SlpA, DR_2310, DR_0505, DR_A0283, and DR_A0282 [69]. Moreover, they hypothesised that in addition to inducing resistance to various threats, the S-layer is also involved in the exchange of compounds and molecules in and out of the cell. This is also supported by evidence showing that protein DR_0774 is involved in extra-cellular transport across the membrane and provides additional structural support to the cell envelope [67]. 

The results of the present study show that HPI and DR_0774 are significantly decreased in abundance. Moreover, three out of the six SDBC proteins, DR_0505, DR_A0283, DR_A0281, are also significantly decreased in abundance, whereas SlpA itself also seems to be decreased in abundance, although it is not below the significance threshold (*q*-value = 0.053). The importance of the S-layer proteins on the integrity of the cell envelope make these results quite surprising, especially since a previous studies performed by Ott et al. [26] demonstrated an increase is S-layer proteins after 48h of simulated microgravity, indicating either an increase in cell envelope size or greater turnover of S-layer proteins, potentially due to damage. It is possible that microgravity initially induces degradation of the S-layer proteins, which might later on trigger an upregulation of S-layer protein expression, thus explaining the discrepancy between the short-term exposure and prolonged exposure to microgravity. The present results show that proteins involved in peptidoglycan biosynthesis, such as murB, murD, murE, and murF, show significant increases in abundance (Figure 7). Moreover, proteins involved in the synthesis of precursors previously shown to be important for peptidoglycan synthesis are also increased in abundance, such as murI (glutamate racemase) [70].

In addition to an upregulation of peptidoglycan biosynthesis pathway components, the present results also show an increased abundance of the protein export (Sec) pathway members. This pathway is the central and essential conserved pathway that is responsible for the export of proteins through the (inner) plasma membrane in Gram-positive and Gram-negative bacteria [71]. Proteins secreted through this pathway can either stay membrane-bound or be secreted to the opposite side of the membrane. Additionally, proteins exported through this pathway can be attached to peptidoglycan, thus this pathway is also involved in the localization of S-layer proteins [72]. The Sec pathway is discussed in detail in the review by Tsirigotaki et al. [71], but briefly summarized herein. Protein export, through this pathway, is conducted in three steps: sorting and targeting, translocation, and maturation and release. Pre-proteins translated in the cytoplasm are targeted to the membrane channel SecYEG by either ffh and its membrane receptor ftsY, or SecA. The protein is translocated through the membrane via the SecYEG channel fuelled by repeated cycles of ATP hydrolysis by SecA, and the proton motive force. Signal peptidases (such as DR_1427) process the protein and it is then released on the other side of the membrane. All these essential components are increased in abundance in *D. radiodurans* cells subjected to microgravity (Figure 5, marked with asterisk), which could imply a potential to increase transmembrane transport during further prolonged response to microgravity.

Another aspect of the results, namely the *tRNA* cluster, could provide further evidence that changes in the cell envelope are being induced by the onset of microgravity. Fifteen out of the 17 proteins in the tRNA cluster are part of the “aminoacyl-tRNA biosynthesis pathway (dra00970)”. Aminoacyl-tRNAs (AA-tRNA) are best known for their role in protein synthesis, where AA-tRNAs deliver amino acids to the ribosomes during the mRNA translation process [73]. However, they have also been shown to be involved in general stress response by attaching amino acids to damaged proteins, thus tagging them for degradation [74,75]. Moreover, AA-tRNAs have been linked with peptidoglycan biosynthesis and lipid membrane transformations and have been directly linked with increased antibiotic resistance in microorganisms (as reviewed in [76,77]). Additionally, the decrease in abundance of ribosomal proteins further suggests that AA-tRNAs might be directed away from their translation function. AA-tRNA upregulation has previously been observed in *D. radiodurans* cells subjected to a simulated space vacuum [30], which could indicate a similar response onset. 

Overall, our results suggest that the cell envelope is being affected by changes in gravity: enrichment of peptidoglycan synthesis pathway, increase in AA-tRNA abundance, decrease in S-layer protein abundance, and increase in sec pathway protein abundance. To elaborate these results further, thorough comprehensive analysis of *D. radiodurans* S-layer is necessary. For instance, subjecting cells to stress stimulus at different time points and employing methods other than -omics techniques, such as structural analysis. Interestingly, it appears that *D. radiodurans* responds to changes in gravitational force with the accumulation of several sensory structures that can further induce major changes in cellular metabolism. The protein DR_A0204 (response regulator) is a part of the two-component signal transduction system (TCS) and shows a 2.5-fold increased abundance in 0 *g*. Proteins in the TCS have been linked to regulation of cell cycle, biofilm formation, and virulence in some bacteria [78,79], and thus could be used by *D. radiodurans* to sense external stress factors. Another protein involved in cell signalling is DR_A0352 (methyl-accepting chemotaxis protein (MCP)), which is increased in abundance 4.22-fold in 0 *g*. MCPs are the main chemoreceptor family in bacteria [80], and are involved in a variety of functions in different species, such as biofilm formation [81], and exopolysaccharide [82] and toxin production [83]. Due to their wide array of potential functions, this *D. radiodurans* MCP could be involved in responding to extracellular stress caused by microgravity. However, further investigation is necessary to understand its exact function.

**Figure 8:**
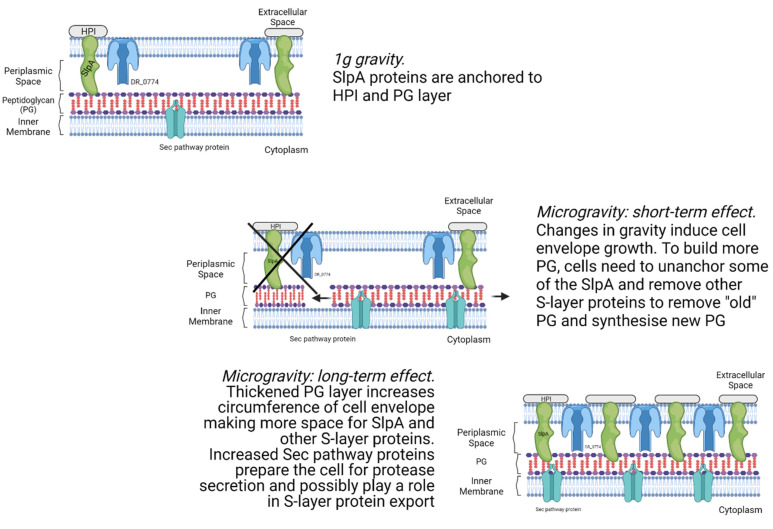
*D. radiodurans* hypothetical cell envelope remodelling with variable abundance of S-layer proteins due to peptidoglycan biosynthesis induced by microgravity during parabolic flight.

Based on the results presented here and published earlier by Ott et al. [26], it is possible to develop a hypothesis as to how *D. radiodurans* regulates its cell envelope remodeling under microgravity conditions. Previous studies have shown that many bacterial species become more resistant to extracellular stressors by increasing cell aggregation and biofilm production when subjected to real or simulated microgravity [84,85,86]. *D. radiodurans* is not known to form biofilms in its natural environment, although mutant strains have shown this ability [87]. Nevertheless, it is possible that *D. radiodurans* also possesses similar mechanisms in response to stress by modulating the thickness of the protective cell envelope. While results after 48 h of microgravity show an increase in S-layer proteins, the immediate response to microgravity presented here shows a decrease in the same proteins. This can possibly be explained by remodeling of cell envelopes and addition of newly synthesized S-layer proteins after long term exposure to microgravity. Bacterial peptidoglycan synthesis has been shown to require removal of “old” glycan chains to synthesize new chains [88]. This could support the hypothesis that expansion of the peptidoglycan layer and subsequently the other cell envelope components cannot occur before some of the anchored S-layer proteins are removed together with the “old” peptidoglycan. After increasing the surface area and/or thickness of the peptidoglycan layer, the S-layer proteins are freshly secreted in larger numbers to match the increased volume/area and would also align well with the results from the simulated microgravity study (hypothesis visualized in Figure 8). This hypothesis could be tested by performing a similar parabolic flight experiment but with more parabolas and timepoints, thus generating a timeline of protein changes. If S-layer proteins do follow a decrease-then-increase pattern, it could provide evidence in favor of this hypothesis.

### 4.3. Does Microgravity Induce DNA Repair?

The presence of the *DNA damage and repair and DNA processing* cluster in the present results might elicit curiosity since one does not immediately correlate seconds in free fall with damage to DNA. Previous studies, however, have indeed shown a link between microgravity and DNA damage during ground-based microgravity experiments. Simulated microgravity experiments on human retinal pigment epithelial cells induced DNA single stand breaks after 48 h [89]. Human bed rest studies, which are used to simulate microgravity on humans, showed an increase in an oxidative DNA damage marker after 60 days [90]. Moreover, the simulated microgravity study performed on *D. radiodurans* by Ott et al. [26] also showed an increase in DNA repair machinery, suggesting damage to DNA after 48h in a clinostat. Thus, this shows that microgravity alone can induce DNA repair. The results here show that in *D. radiodurans* DNA repair and stress response proteins are differentially abundant after short-term exposure to free fall. Overall, 17 proteins showed significant increases in abundance in 0 *g* compared to GC. DNA-directed polymerase (DR_1707 or PolA) and DNA ligase (DR_2069 or LigA) show an increase of more than twofold and exonuclease ABC subunit B (DR_2275 or UvrB) and transcription-repair coupling factor (DR_1532 or Mfd) show an increase of 1.7-fold and 1.59-fold, respectively. These four proteins are part of the nucleotide excision repair (NER) pathway (dra03420), which is responsible for the recognition and repair of DNA lesions that can be caused by chemicals, radiation, and ROS [91]. Three of these proteins (excluding UvrB) were also increased after 48h in simulated microgravity [26]. Other proteins that have previously been implicated in DNA repair in *D. radiodurans* are significantly increased in abundance, such as RecA, which has a role in genome reconstitution after severe damage [92]. Interestingly, a decrease in abundance of some proteins involved in DNA repair and processing was also noted. The protein with the most decreased abundance is the extracellular nuclease (DR_B0067), which is the only extracellular nuclease encoded by the *D. radiodurans* genome [93]. In their study, Li et al. [93] investigated this nuclease and showed that DR_B0067 mutants had impaired resistance to hydrogen peroxide and that this protein played an important role in oxidative stress resistance by breaking down extracellular DNA. As shown previously, nucleosides and bases can protect proteins from ROS-induced damage [94], thus this degradation of DNA would then provide more nucleosides and more ROS protection [93]. Therefore, it is rather unclear why this protein would decrease in abundance. It could possibly be explained by the absence of extracellular DNA; however, this cannot be confirmed due to a lack of data. Therefore, future studies should investigate the specific triggers of DR_B0067 altered abundance in microgravity. 

The *transcription* cluster of proteins also shows that the pull of DNA repair enzymatic activities is enriched, potentially preparing for a long-term molecular response. The protein with the highest increase in abundance in 0 *g* in this category was hypothetical protein DR_1872. There is no experimental information about its function besides its GO BP term and 100% identity to a *Deinococcus wulumuqiensis* transcriptional regulator. However, it showed a 3.88-fold increase in abundance in 0 *g* compared to GC. Thus, DR_1872 is potentially a good target for future investigations into short-term responses to microgravity in *D. radiodurans*. Among the other proteins with significantly increased abundance, three out of four subunits of RNA polymerase (RNAP) are present. RNA polymerase is a protein complex involved in the synthesis of messenger RNA and non-coding RNA from the DNA template strand. Additionally, transcription factor Rho and transcription termination/antitermination protein NusA show increased abundance in 0 *g*. In bacteria, these proteins are all linked by the transcription-coupled DNA repair (TCR). TCR is a DNA repair mechanism that is present in prokaryotes as well as eukaryotes [95]. In TCR, RNAP elongating RNA stops at DNA lesions. This event recruits the NER-machinery to the DNA lesion and initiates the DNA repair process, thus RNAPs are effectively DNA damage sensors. The aforementioned Mfd removes the stalled RNAP from the lesion by pushing it forward and recruits further NER components [96]. What is more interesting, a recent study showed that transcription factor Rho might carry out a similar function to Mfd, thus further facilitating DNA repair [97]. Additionally, an Mfd-independent TCR pathway has been shown to involve the NusA protein, where it works with UvrD to pull the RNAP, revealing the lesion [98]. 

Decreased abundance proteins in the *Transcription* cluster also show some interesting patterns. The protein with the most decreased abundance is the CSD family cold shock protein (DR_0907), showing a decrease of approximately 25-fold in 0 *g*. The study by Anaganti et al. [99] investigated the importance of DR_0907 on *D. radiodurans* cellular functions and found that DR_0907 mutants showed a wide variety of affected cellular functions. They showed a downregulation of proteins involved in tRNA aminoacylation, DNA metabolism, nucleotide sugar metabolism, and TCA cycle, whereas oxidative stress defence and pyrimidine ribonucleotide metabolism were upregulated. Our result might indicate an inverse link between this protein and the response to microgravity, but further investigations are necessary to understand the exact role in this response. Other decreased abundance proteins in this cluster include three TetR family transcriptional regulators. This family of transcriptional regulators is one of the largest families of transcription factors in bacteria [100] and is generally associated with the repression of associated genes. TetR transcription factors are known to regulate efflux pump regulation, amino acid metabolism, and biofilm formation [100]. It is possible that the TetR transcription factors play an important role in the long-term *D. radiodurans* response to microgravity, and this role should be further investigated in future studies. *D. radiodurans*’ ability to survive extreme environmental changes can possibly be explained by its high tolerance and/or resistance to DNA damage. The fast changes in DNA repair machinery abundances likely explain these traits, as an early response to mitigate DNA damage and repair it lead to higher chances of survival. Therefore, it is possible that the response observed in the *transcription* functional cluster is evidence that environmental stress leads to *D. radiodurans* preparing for DNA damage within seconds of onset. However, a proteomics study alone is not sufficient to determine the sequence of events leading to transcriptional regulation. Conducting a subsequent transcriptomics study on the short-term response is necessary to reveal a decrease or increase in overall transcription levels of these proteins and could provide further evidence as to which process is taking place.

The present results show an increase in the TCA cycle enzymes, as well as enzymes involved in the pentose phosphate pathway (Table 2). A previous study on the methicillin-resistant *Staphylococcus aureus* has linked increased energy metabolism, and especially upregulation of the TCA cycle, to increased production of ROS [101]. In our study, an increase in the TCA cycle enzyme abundances are observed, as well as for enzymes involved in the pentose phosphate pathway (Table 2). Moreover, our results also indicate that the cell envelope might be subjected to additional stress, which could further destabilize the respiratory chain enzymes. This destabilization and increase in TCA cycle activity possibly leads to increased ROS production. Results also show that multiple proteins involved in protein folding and general stress response are increased in abundance (Table 2), potentially indicating that proteins are sustaining damage in microgravity. An increase in oxidative stress proteins from the *stress response* cluster further supports increased ROS production. Thioredoxin reductase (DR_1982) shows a 2.24-fold increase in 0 *g*. This protein is an important antioxidant, defending DNA from oxidative damage caused by oxygen metabolism (i.e., ROS) through redox cycles involving NADPH and ROS-scavenging species [102]. Moreover, it is part of the thioredoxin system and is required for the reduction of ribonucleotides to deoxyribonucleotides, thus playing a role in DNA synthesis and repair [103]. In this cluster, multiple proteins with oxidoreductase activity show significant increases in abundance, indicating potential destabilization of redox homeostasis. However, this cluster also contains many decreased-abundance stress response proteins with similar properties, suggesting complex oxidative stress management in *D. radiodurans* that does not depend on one type of element.

It is difficult to attribute the observed changes in protein abundance to altered gene expression, as minimal time needed for bacterial signalling, transcription, and translation significantly exceeds 10 s exposure to microgravity aboard of parabolic flight. Our presented findings on proteome changes in *D. radiodurans* under short-term microgravity could rather be related to a difference in protein stability, protein degradation and consequentially changes in protein ratios. Protein degradation processes have been shown to be triggered by short-term microgravity in primary human macrophages aboard the ISS [104]. The authors suggested ubiquitin-related protein degradation as a starting protein degradation during a stress response and the most likely source of increased amino acid levels after short-term microgravity [104]. Additionally, protein degradation as a cellular stress response is known to be reflected by the microgravity-affected ubiquitin pools during parabolic rocket flights [105]. Protein degradation commonly occurs as a stress response, e.g., during starvation, depletion of nutrients or in oxidative stress conditions, when reactive oxygen species damage proteins [106]. Previous investigations showed that ROS production is highly dependent on changes in the gravity environment and reacts to gravitational stimuli within ~20 s of parabolic flight [107]. ROS metabolism might contribute to the regulation of protein degradation in microgravity to maintain energy homeostasis and help with nutrient utilization to rapidly adapt to the stress caused by the extraordinary circumstances. We propose that altered protein degradation could represent the underlying explanation of the observed proteomic changes in our study. This degradation process could be generated by a specific response to a different cellular environment and regulated by the general cellular protease/hydrolase system, which has been observed in *D. radiodurans* when grown in simulated microgravity [26]. Nevertheless, further investigations are required to better understand the gravity-sensitive nature of protein degradation and its functional mechanism. Further omics-assisted studies could elucidate whether the antibiotics as translation inhibitors can alter differential expression patterns during microgravity.

## 5. Conclusions

**Figure 9:**
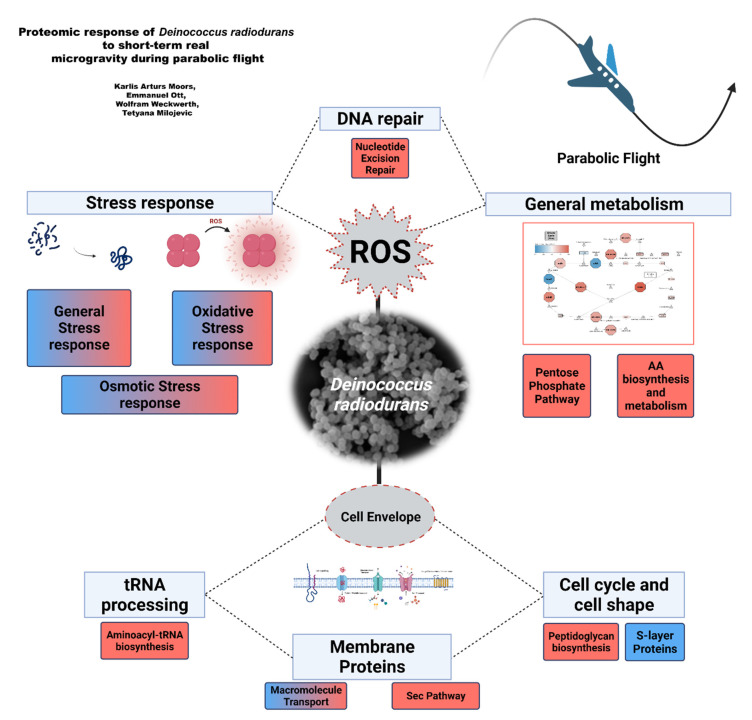
Multiple functional categories of differentially abundant proteins of *D. radiodurans* during parabolic flight. Red color indicates significant increase in protein abundance in microgravity in the respective functional cluster. Blue color indicates significant decrease in protein abundance in microgravity in the respective functional cluster.

Understanding the molecular alterations occurring in bacteria due to changes in gravity is important in the context of future space missions, as microgravity is an important variable in outer space. The present study was performed to elucidate the changes to the *D. radiodurans* proteome that occur after exposure to short-term real microgravity aboard a parabolic flight. Our investigations showed that increased gravity had no significant effect on the *D. radiodurans* proteome, whereas 10 s in microgravity (0 *g*) elicited a pronounced change in multiple functional categories (summarized in Figure 9). The results provided evidence that the *D. radiodurans* cell envelope undergoes changes, highlighted by increased abundance of proteins involved in peptidoglycan biosynthesis and decreased abundance of S-layer proteins, which might indicate that microgravity induces stress to the cell envelope. The increased aminoacyl-tRNA abundance further supports this. Based on the present findings and our previous work, we hypothesized that *D. radiodurans* require brief degradation of ‘’old’’ peptidoglycan and S-layer proteins to generate a higher number of S-layer proteins, which are crucial in the response against environmental stress (Figure 8). General metabolism proteins also showed differences in abundance, with increased-activity energy metabolism pathways potentially showing that increased energy is needed in response to microgravity. The elevated oxidative stress response and observed increase in abundance of DNA repair proteins might suggest that the bacterium is preparing to mitigate an increase in ROS production, potentially originating from an increase in energy metabolism and changes in the cell envelope. ROS-induced production is a well-known factor that contributes to cellular stress and degradation in astronauts during spaceflight. Recent research has been aimed at mitigating these negative effects, and proposals for the development of antioxidant cocktails have been made [108]. The results of the current research and the overall radiation-resistant traits of *D. radiodurans* suggest that this organism has an extremely rapid and efficient response to dealing with increased ROS-production. Therefore, it is possible that understanding the intricacies of this early reaction in *D. radiodurans* can aid in the efforts to provide a safer environment for humans in space. Overall, these results, together with those of our previous study on prolonged exposure to simulated microgravity, provide insights into the timeline of molecular changes in the *D. radiodurans* response to microgravity. Although our findings indicate a massive response to microgravity, further structural investigation of the cell envelope, and a targeted metabolomics study is necessary to validate the findings and elucidate the role of individual proteins in the overall response. Additionally, future experiments should focus on prolonging the microgravity duration, increasing the number of multiple time-points and flight replicates, and adding other -omics measurements, such as transcriptomics and metabolomics, to obtain a comprehensive view of the molecular processes affected by microgravity. Moreover, other ground-based methods, such as drop towers or sounding rockets, could be used in the future to elucidate any potential method-specific idiosyncratic responses and further clarify the response of *D. radiodurans* to changes in gravitational acceleration.

## Figures and Tables

**Table 1:** *D. radiodurans* proteins measured during parabolic flight with significantly different increased and decreased abundance unique to the 0 *g* vs. FC contrast (not significant in the 0 *g* vs. GC contrast). First three columns show proteins with increased abundance; last three columns show proteins with decreased abundance.

Protein Name	Gene Name	Log2 Fold Change	Protein Name	Gene Name	Log2 Fold Change
**General Metabolism**			**General Metabolism**		
Glutamate 5-kinase	proB	3.52	Cytochrome-related protein	DR_0429	−0.54
Alanine dehydrogenase	DR_1895	0.64	3-isopropylmalate dehydratase small subunit 2	leuD2	−0.48
Aspartokinase	DR_1365	0.56	Purine-nucleoside phosphorylase	DR_2166	−0.47
Glycogen synthase	glgA	0.46	UDP-N-acetyl-D-mannosaminuronic acid transferase, putative	DR_1645	−0.43
Acetyl-CoA acetyltransferase	DR_A0053	0.44	Lipopolysaccharide biosynthesis protein, putative	DR_A0043	−0.40
Citrate lyase subunit beta-like protein	DR_1240	0.38	dTDP-glucose 4,6-dehydratase	DR_A0041	−0.38
3-oxoacyl-[acyl-carrier-protein] synthase 2	DR_1941	0.35	Succinate--CoA ligase [ADP-forming] subunit alpha	sucD	−0.34
Histidinol dehydrogenase	hisD	0.31			
**Membrane Proteins**			**Membrane Proteins**		
Uncharacterized protein	DR_0458	0.44	Sodium extrusion protein NatA	DR_0927	−0.44
**DNA damage and repair, DNA processing**			**DNA damage and repair, DNA processing**		
Replicative DNA helicase	DR_0549	0.56	Probable chromosome 1-partitioning protein ParB	parB1	−0.32
DNA gyrase subunit A	gyrA	0.54			
DNA topoisomerase 1	topA	0.41			
Endonuclease MutS2	mutS2	0.36			
**Transcription**			**Transcription**		
Transcriptional regulator MraZ	mraZ	0.34	Probable transcriptional regulatory protein DR_2548	DR_2548	−1.09
			DNA-binding response regulator	DR_0432	−0.55
			Transcription termination/antitermination protein NusG	nusG	−0.40
**Cell cycle and cell shape**			**Cell cycle and cell shape**		
UDP-N-acetylmuramate--L-alanine ligase	murC	0.75			
**Stress Response**			**Stress Response**		
Thiol:disulfide interchange protein	DR_0189	0.46	60 kDa chaperonin	groL	−0.65
Leucyl aminopeptidase, putative	DR_0717	0.38	Phosphinothricin acetyltransferase	DR_1182	−0.55
Carboxyl-terminal protease, putative	DR_1491	0.33	Chaperone protein DnaK	dnaK	−0.50
			N-acetyltransferase domain-containing protein	DR_0653	−0.48
			Lon protease	DR_0349	−0.44
			Putative phosphoenolpyruvate synthase regulatory protein	DR_1728	−0.35
**tRNA processing**			**tRNA processing**		
			Methylenetetrahydrofolate--tRNA-(uracil-5-)-methyltransferase TrmFO	trmFO	−0.71
**Translation, Ribosomes and rRNA**			**Translation, Ribosomes and rRNA**		
			Glutamyl-tRNA(Gln) amidotransferase subunit A	gatA	−0.52
			30S ribosomal protein S2	rpsB	−0.49
			Elongation factor Ts	tsf	−0.42
			Ribosome-recycling factor	frr	−0.40
**Unknown**			**Unknown**		
KAP NTPase domain-containing protein	DR_C0009	1.36	Uncharacterized protein	DR_2563	−0.85
Uncharacterized protein	DR_1256	1.35	Uncharacterized protein	DR_1252	−0.73
Uncharacterized protein	DR_1331	1.32	Uncharacterized protein	DR_0389	−0.67
Metallophos domain-containing protein	DR_1119	1.07	Heat shock protein, HSP20 family	DR_1114	−0.66
Uncharacterized protein	DR_0360	0.91	Uncharacterized protein	DR_0994	−0.46
Ferripyochelin-binding protein	DR_2089	0.73	Glyoxalase-like_dom domain-containing protein	DR_2014	−0.44
Uncharacterized protein	DR_1018	0.63	Lipopolysaccharide biosynthesis protein, putative	DR_0444	-0.39
GHL10 domain-containing protein	DR_A0207	0.53	DUF1990 domain-containing protein	DR_A0230	−0.38
Uncharacterized protein	DR_1773	0.40	zf-RING_7 domain-containing protein	DR_0291	−0.36
Uncharacterized protein	DR_A0190	0.37	Site-determining protein	DR_0752	−0.34
Propionyl-CoA carboxylase, beta subunit, putative	DR_1542	0.36	DUF11 domain-containing protein	DR_0685	−0.33
Uncharacterized protein	DR_0574	0.33	Uncharacterized protein	DR_2057	−0.30
Uncharacterized protein	DR_A0022	0.32	Chromosome partitioning ATPase, putative, ParA family	DR_A0001	−0.27

**Table 2:** Significant D. *radiodurans* proteins measured during parabolic flight with significantly increased abundance in the 0 *g* vs. GC contrast within the pentose phosphate pathway (PPP), as well as DNA damage and repair, DNA processing, transcription, and stress response clusters.

Protein Name	Gene Name	Log2 Fold Change
**Pentose Phosphate Pathway (PPP)**		
phosphopentomutase	DR_2135	1.28
glucose-6-phosphate 1-dehydrogenase	DR_1596	0.80
carbohydrate kinase	DR_1525	0.61
glucose-6-phosphate isomerase	pgi	0.52
2-deoxyribose-5-phosphate aldolase	DR_1205	0.49
**DNA Damage and Repair, DNA Processing**		
DNA ligase	DR_2069	1.04
DNA-directed DNA polymerase	DR_1707	1.01
hypothetical protein	DR_0428	0.83
exconuclease ABC subunit B	DR_2275	0.77
Mrr restriction system protein	DR_0508	0.77
hypothetical protein	DR_0326	0.68
transcription-repair coupling factor	DR_1532	0.67
ParB family chromosome partitioning protein	DR_A0002	0.58
Beta sliding clamp	DR_0001	0.54
exonuclease SbcC	DR_1922	0.54
hypothetical protein	DR_2235	0.54
Ribonucleoside-diphosphate reductase (EC 1.17.4.1)	DR_B0108	0.48
MTS domain-containing protein	DR_0914	0.44
Holliday junction ATP-dependent DNA helicase RuvB (EC 3.6.4.12)	RuvB	0.39
Ribonucleoside-diphosphate reductase (EC 1.17.4.1)	DR_2374	0.35
Protein RecA (Recombinase A)	RecA	0.31
**Transcription**		
hypothetical protein	DR_1872	1.96
transcription termination factor Rho	rho	1.08
DNA-directed RNA polymerase subunit beta’	DR_0911	1.02
Lpr/AsnC family transcriptional regulator	DR_0200	0.93
Transcriptional regulator, HTH_3 family	DR_2574	0.79
DNA-directed RNA polymerase subunit alpha	DR_2128	0.73
DNA-directed RNA polymerase subunit beta	rpoB	0.53
Transcription termination/antitermination protein NusA	NusA	0.52
Bifunctional protein PyrR [Includes: Pyrimidine operon regulatory protein; Uracil phosphoribosyltransferase (UPRTase)]	PyrR	0.38
magnesium protoporphyrin chelatase	DR_2594	0.34
**Stress Response**		
*Osmotic Stress*		
HAMP domain-containing protein	DR_1829	0.92
*Oxidative Stress*		
NADH-dependent flavin oxidoreductase	DR_2190	1.55
thioredoxin reductase	DR_1982	1.17
thiol-specific antioxidant protein	DR_2242	0.85
LuxA-like protein	DR_0611	0.79
short chain dehydrogenase/reductase family oxidoreductase	DR_1938	0.79
3-hydroxyisobutyrate dehydrogenase	DR_0499	0.74
Uncharacterized protein	DR_1002	0.58
Oxidoreductase, short-chain dehydrogenase/reductase family	DR_0113	0.54
zinc-containing alcohol dehydrogenase	DR_A0005	0.51
Dihydrolipoyl dehydrogenase	DR_2370	0.43
*General Stress*		
oligoendopeptidase F	DR_2055	1.26
prolyl endopeptidase	DR_2503	1.19
proline iminopeptidase-like protein	DR_0654	1.17
oligoendopeptidase	DR_1627	1.15
hypothetical protein	DR_2363	1.11
carboxypeptidase G2	DR_2493	0.91
hypothetical protein	DR_0985	0.87
metalloprotease	DR_0617	0.86
ATP-dependent Clp protease, ATP-binding subunit ClpB	ClpB	0.83
NAD-dependent protein deacylase	cobB	0.79
oligopeptidase A	DR_1659	0.70
Protein GrpE (HSP-70 cofactor)	GrpE	0.70
cyclophilin-type peptidyl-prolyl cis-trans isomerase	DR_2542	0.67
hypothetical protein	DR_1832	0.60
Aminoglycoside N(3)-acetyltransferase	DR_2034	0.52
ATP-dependent protease LA	Lon	0.51

## Data Availability

The mass spectrometry proteomics data have been deposited in the ProteomeXchange Consortium (http://proteomecentral.proteomexchange.org (accessed on 19 December 2021)) via the PRIDE partner repository [109] with the dataset identifier PXD027236. R code and processed proteomics data can be found in https://github.com/kamoors/ParabolicFlight (accessed on 19 December 2021).

## Supplementary Materials

The following are available online at: https://www.mdpi.com/article/10.3390/life12010023/s1, Figure S1: Schematic of the custom-made injector used during the parabolic flight to inject overnight cultures of *D. radiodurans* in methanol (MeOH)., Figure S2: Illustration of sampling points during the parabolic flight. Figure S3: PCA plots using data of the 1200 proteins and 11 samples used for analysis before (top) and after (bottom) removal of sample GC_4. Table S1: Statistical analysis output data. Table S2: List of proteins with significantly decreased abundance in *D. radiodurans* exposed to microgravity during parabolic flight from the 0 *g* vs. GC contrast. Proteins are grouped by functional category. Table S3: Statistical analysis output data.
