# Supplementary material for: Proteomic Response of Deinococcus radiodurans to Short-Term Real Microgravity during Parabolic Flight Reveals Altered Abundance of Proteins Involved in Stress Response and Cell Envelope Functions
Source: Life (Basel). 2021 Dec 24;12(1):23. doi: 10.3390/life12010023 (PMC8779699; doi:10.3390/life12010023)
Supplement: Supplementary file 1 [file life-12-00023-s001.zip › Supplementary Figures.pdf]

life

MDPI

Supplementary Materials: Proteomic Response of Deinococcus Radiodurans to Short-term Real Microgravity During Parabolic Flight Reveals Altered Abundance of Proteins Involved in Stress Response and Cell Envelope Functions

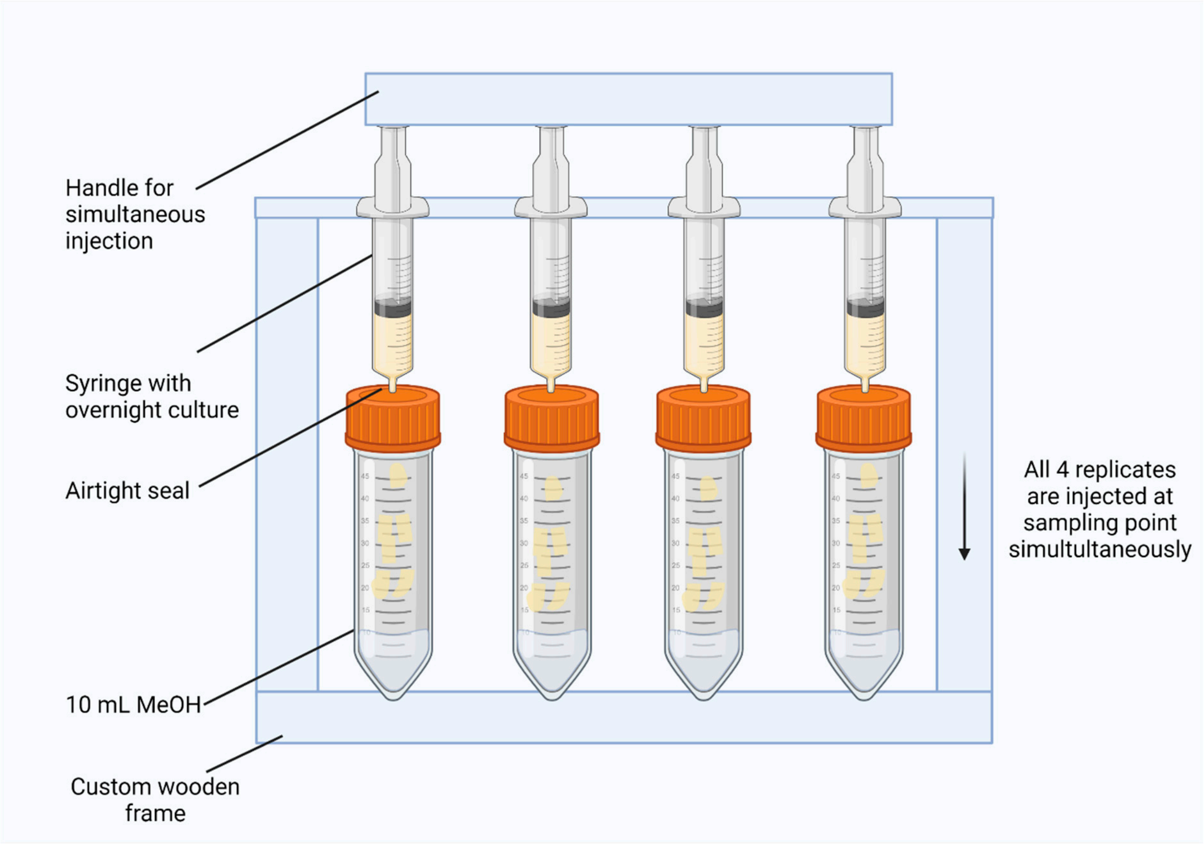

Figure S1. Schematic of the custom-made injector used during the parabolic flight to inject overnight cultures of *D. radiodurans* in methanol (MeOH).

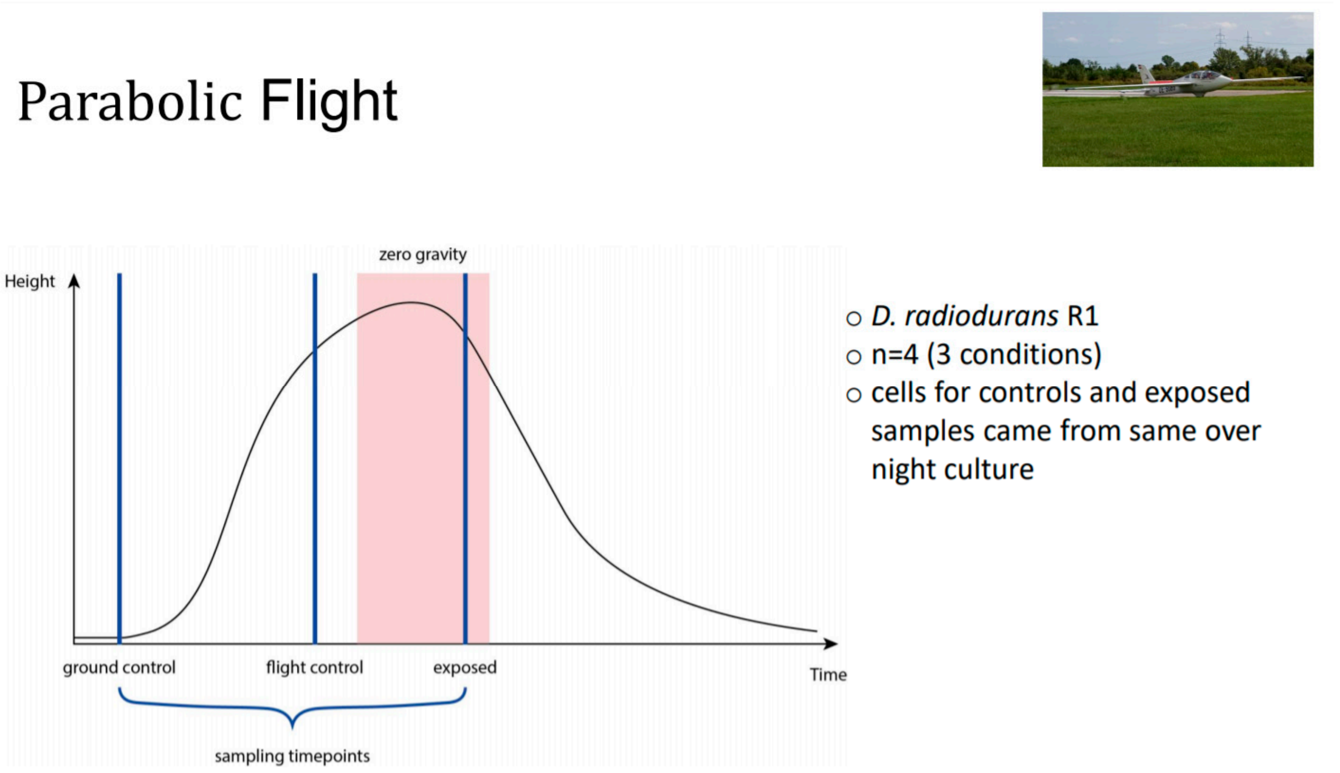

Figure S2. Illustration of sampling points during the parabolic flight.

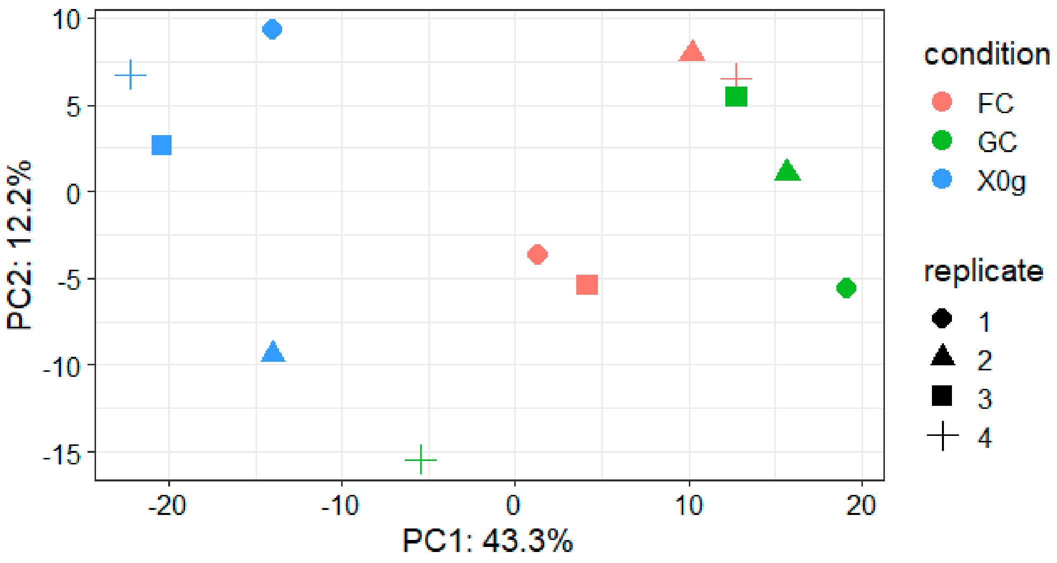

(a)

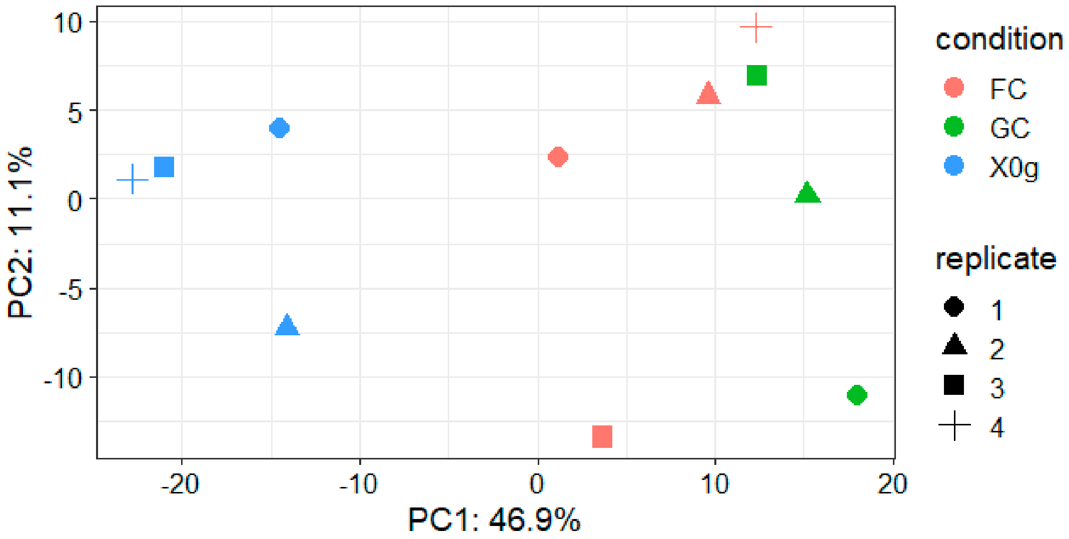

(b)

Figure S3. PCA plots using data of the 1200 proteins and 11 samples used for analysis before (**a**) and after (**b**) removal of sample GC4.
